# Development and validation of a quantitative choline food frequency questionnaire for use with drinking and non-drinking pregnant women in Cape Town, South Africa

**DOI:** 10.1186/s12937-018-0411-5

**Published:** 2018-11-22

**Authors:** R. Colin Carter, Sandra W. Jacobson, Sharmilah Booley, Baheya Najaar, Neil C. Dodge, Lori J. Bechard, Ernesta M. Meintjes, Christopher D. Molteno, Christopher P. Duggan, Joseph L. Jacobson, Marjanne Senekal

**Affiliations:** 1 Institute for Human Nutrition and Departments of Emergency Medicine and Pediatrics, Columbia University Medical Center, 3959 Broadway, CHN-1-116, New York, NY 10032 USA; 2 Department of Psychiatry and Behavioral Neurosciences, Wayne State University School of Medicine, Detroit, USA; 3 Department of Psychiatry and Mental Health, University of Cape Town Faculty of Health Sciences, Cape Town, South Africa; 4 Department of Human Biology, University of Cape Town Faculty of Health Sciences, Cape Town, South Africa; 5 Division of Gastroenterology, Hepatology, and Nutrition, Boston Children’s Hospital, Boston, USA

**Keywords:** Choline, Food frequency questionnaire, Nutrition, Alcohol consumption during pregnancy, Diet, Fetal alcohol spectrum disorders, Prenatal alcohol exposure

## Abstract

**Background:**

Although animal and human studies have demonstrated interactions between dietary choline and fetal alcohol spectrum disorders, dietary choline deficiency in pregnancy is common in the US and worldwide. We sought to develop and validate a quantitative food frequency questionnaire (QFFQ) to estimate usual daily choline intake in pregnant mothers.

**Methods:**

A panel of nutrition experts developed a Choline-QFFQ food item list, including sources with high choline content and the most commonly consumed choline-containing foods in the target population. A data base for choline content of each item was compiled. For reliability and validity testing in a prospective longitudinal cohort, 123 heavy drinking Cape Coloured pregnant women and 83 abstaining/light-drinking controls were recruited at their first antenatal clinic visit. At 3 prenatal study visits, each gravida was interviewed about alcohol, smoking, and drug use, and administered a 24-hour recall interview and the Choline-QFFQ.

**Results:**

Across all visits and assessments, > 78% of heavy drinkers and controls reported choline intake below the Dietary Reference Intakes adequate intake level (450 mg/day). Women reported a decrease in choline intake over time on the QFFQ. Reliability of the QFFQ across visits was good-to-acceptable for 2 of 4 group-level tests and 4 of 5 individual-level tests for both drinkers and controls. When compared with 24-hr recall data, validity of the QFFQ was good-to-acceptable for 3 of 4 individual-level tests and 3 of 5 group-level tests. For controls, validity was good-to-acceptable for all 4 individual-level tests and all 5 group-level tests.

**Conclusions:**

To our knowledge, this is the first quantitative choline food frequency screening questionnaire to be developed and validated for use with both heavy and non-drinking pregnant women and the first to be used in the Cape Coloured community in South Africa. Given the high prevalence of inadequate choline intake and the growing evidence that maternal choline supplementation can mitigate some of the adverse effects of prenatal alcohol exposure, this tool may be useful for both research and future clinical outreach programs.

## Background

Fetal alcohol spectrum disorders (FASD) comprise a continuum of alcohol-related neurodevelopmental disorders ranging from the most severely affected children with fetal alcohol syndrome (FAS) to nonsyndromal children who also exhibit neurocognitive and/or behavioral deficits but may lack the facial features or growth deficits seen with FAS [1, 2]. Worldwide, a significant number of women drink heavily during pregnancy despite public health advisories and psychosocial interventions [3–5]. In the Western Cape Province of South Africa, where rates of heavy drinking during pregnancy are endemically high among women from the Cape Coloured (mixed ancestry) community [6–8], the prevalence of FAS is as high as 80 per 1000 [9].

A growing body of studies in FASD animal models has demonstrated that optimal maternal choline status can mitigate some of the teratogenic effects of alcohol [7, 10–16]. Thomas and colleagues have shown protective effects of pre- and postnatal choline supplementation on hippocampal development and related neurobehavioral outcomes in rats [10, 11], including reversal of alcohol-related deficits in eyeblink conditioning, which we have also shown to be profoundly affected in children prenatally exposed to alcohol [7, 12, 13]. We have recently extended these findings to humans in an exploratory randomized, double-blind, controlled trial, which demonstrated that high-dose choline supplementation initiated early in pregnancy can mitigate adverse effects of heavy drinking on infant eyeblink conditioning, cognition, and post-natal growth [14, 15].

Choline is an essential nutrient that is a constituent of the neurotransmitter acetylcholine and a precursor to phosphatidylcholine and sphingomyelin, which are major components of cell membranes and play an important role in cell membrane integrity, trans-membrane signaling, and triglyceride turnover from the liver and blood [16]. In addition, it serves as a methyl-group donor needed for homocysteine metabolism and DNA methylation, a critical mechanism in epigenetic processes that has been implicated in alcohol teratogenesis. Despite the fact that choline can be produced endogenously, it is classified as an essential nutrient [17, 18], and dietary intake, principally from eggs, liver, wheat germ, and milk, is imperative to meet physiological needs. The demand for choline is especially high during pregnancy, when it is actively transported to the fetus against a concentration gradient [19] and depletes maternal stores [20, 21]. In a recent randomized, double-blind, controlled trial comparing two 3^rd^-trimester dietary regimens – 480 mg choline/day (just above the adequate intake level of 450 mg/day [19]) vs. 930 mg/day (just over twice adequate intake level), the higher choline intake arm was associated with faster infant processing speed through age 13 mo [22].

We have recently demonstrated that, although the diet and body composition of heavy drinking pregnant women is similar to that of abstaining/light-drinking pregnant women in socio-economically disadvantaged areas in Cape Town, South Africa, in repeated 24-hr dietary recall interviews, almost 90% of the women reported inadequate intake of choline [23]. High rates of inadequate choline intake during pregnancy have also been reported in the U.S. (90%) [24, 25], Canada (77%) [26], and New Zealand (84%) [27]. These studies emphasize the importance of assessment of dietary choline intake in groups at risk for inadequate intake, such as pregnant women, to inform dietary choline interventions, as well as the use of choline supplements. The use of repeated 24-hour recall interviews to assess usual daily intake of micronutrients has been validated in the U.S. and in resource-poor settings but is both labor and time-intensive [28]. One alternative is to use a dietary intake assessment method that focuses specifically on quantification of choline content using a quantitative nutrient-indicated food frequency questionnaire (QFFQ). This methodology is increasingly applied in assessment of usual intake of single nutrients (e.g., vitamin D [29], iron [30], and vitamin K [31].

We developed a quantitative choline food frequency questionnaire (Choline-QFFQ) to estimate usual dietary choline intake (mg/day) by participants in our Cape Town randomized clinical trial conducted to assess feasibility and efficacy of a maternal choline supplementation intervention conducted with heavy drinking women during pregnancy [14, 15]. The current study combines data from this trial and from our larger prospective longitudinal cohort on the effects of prenatal alcohol exposure on development that included both heavy drinking and abstaining/light-drinking pregnant women from the same community as the women in the trial [8, 32]. Our aims were (1) to develop a Choline-QFFQ that can be used to estimate usual daily intake of dietary choline and (2) to test its reliability and validity in both heavy drinkers and abstainers/light-drinkers.

## Methods

### Development of the Choline-QFFQ

The Choline-QFFQ food item list was developed by a panel of three nutrition experts in the Division of Human Nutrition, University of Cape Town, including M.S, S.B, and B.N., in which food sources with high choline content, as well as the most commonly consumed foods in the target population were included. The food sources list was derived from Nel and Steyn [33] and multiple unpublished field studies completed by post-graduate students in dietetics in Cape Town, South Africa. In addition to high choline-content foods, commonly consumed food sources with moderate or low-choline content were included in the final food list, as frequent consumption of such foods may also make a considerable contribution to choline intake. Lewis et al. [26] demonstrated that vegetables, baked products and fruits that are moderate to low sources of choline provided 8.5%, 7.7% and 6.6%, respectively, of total choline intake versus 12.2% provided by eggs and 11.1% by meat, which contain high levels of choline. The final food list was comprised of 10 groups, including beef (organ, mince, patty, chops and braised beef), lamb (chops and stew), chicken (fried or roasted in different cuts, giblets and liver), processed meats (salami, ham, polony, viennas and frankfurters), fish (canned sardines and canned pilchards), eggs (boiled or fried), legumes (baked beans, lentils, sugar beans, soy beans), vegetables (cauliflower, broccoli, corn/mealies, peas, mixed vegetables, spinach, gem squash, pumpkin, potatoes – fried or mashed with added milk), fruit (banana), dairy (milk drink, milk added to cereal or in tea/coffee and yoghurt) and other items (beer, chocolate cake, tomato sauce/ketchup and peanut butter). Participants were requested to report the number of times a particular food item had been consumed during the past week, either number of times per day, if consumed daily, or otherwise number of times in the past week.

**Fig. 1:**
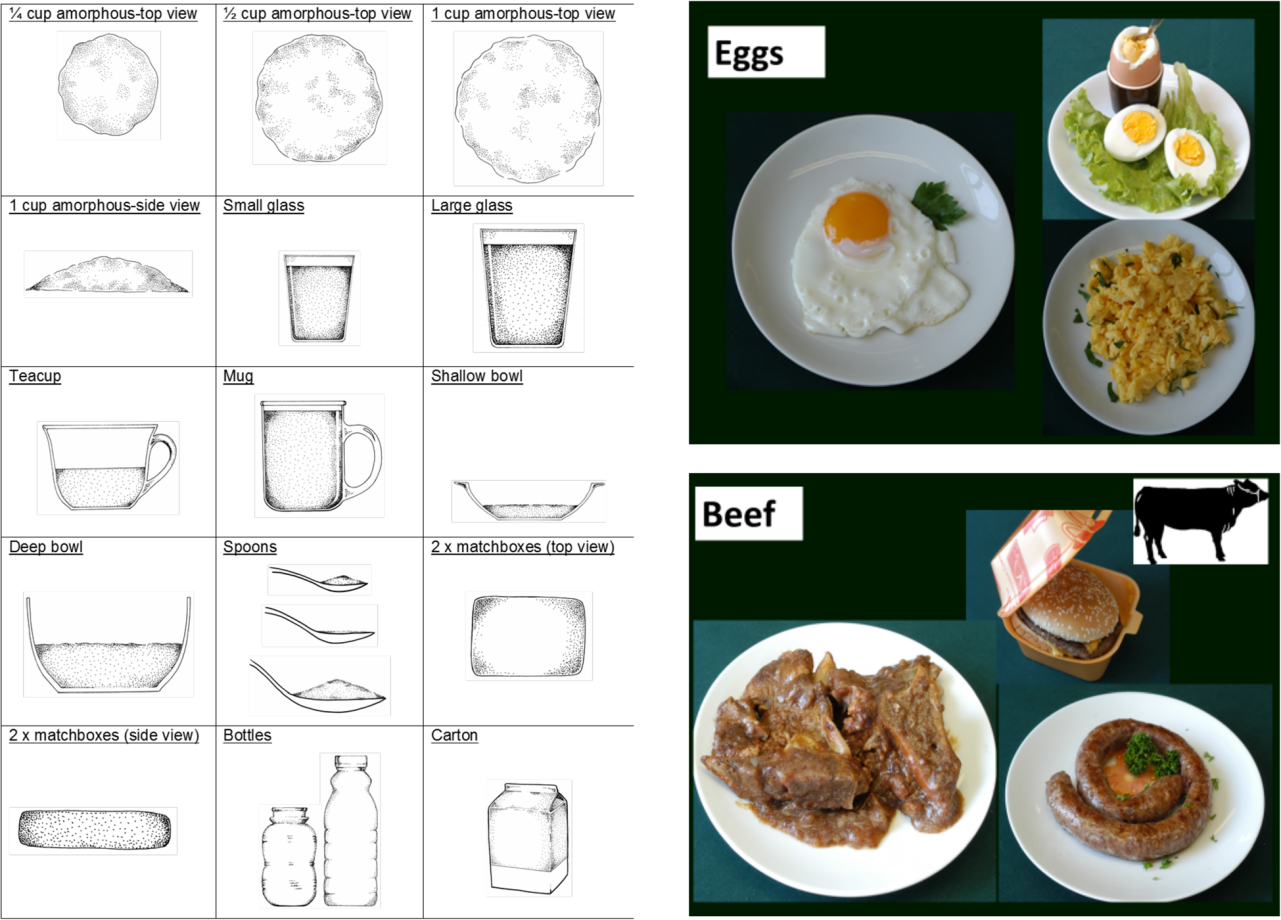
Pictures and portion size props used from the Dietary Assessment and Education Kit [34]

The picture sort method was used for administration of the Choline-QFFQ, whereby participants were asked to sort picture cards of food items included in the food list into two piles, those consumed during the past week and those not consumed during the past week. Interviewers, either a registered dietician or a research assistant with extensive training in dietary interviewing by M.S., then proceeded to quantify the frequency of intake and typical portion size for items that had been consumed during the past week. Portion size estimation was conducted using pictures and portion size props (Fig. 1) included in the Dietary Assessment and Education Kit (DAEK) [34]. The Choline-QFFQ was pilot-tested with 24 women of child-bearing age from the same community as the main cohort in this study. Ease of procedure, face validity, and acceptability among subjects were excellent.

### Study Participants for Reliability and Validity Testing

This study included pregnant women from the Cape Coloured (mixed ancestry) community who were recruited to participate in the choline clinical trial [14, 15] or the prospective longitudinal study [23] between October 2011 and December 2015. Women were recruited at their first visit to one of three antenatal midwife obstetric units that serve socioeconomically-disadvantaged communities in Cape Town (see [15, 16, 27] for further subject eligibility, recruitment, retention, and sample size calculation details). Each mother was interviewed at screening (*M* = 21.1 weeks gestation, SD = 6.1) regarding her alcohol consumption both at time of conception and recruitment and again at 4 and 12 weeks after recruitment, by a research nurse using the timeline follow-back interview (TLFB; [35]) adapted for use with women in this community [7, 32]. Any woman averaging at least 1.0 oz absolute alcohol (AA)/day (1 oz AA~2 standard drinks) or reporting binge drinking (≥2.0 oz AA/drinking occasion) was invited to participate in the study. Women initiating antenatal care who drank only minimally and did not binge drink were invited to participate as abstainers/light drinkers. Exclusion criteria included age <18 years, HIV infection, and pharmacologic treatment for medical conditions, including diabetes, hypertension, epilepsy, or cardiac problems. All women who reported drinking during pregnancy were advised to stop or reduce their intake, and women were offered a referral for substance abuse treatment programs. Consent and interviews were conducted in Afrikaans or English, depending on the mother’s preference.

### Reliability and validity testing of the Choline-QFFQ

Study visits occurred at recruitment and again 4 and 12 weeks later. Both the test method (Choline-QFFQ) and reference method (24-hr recall) were administered at each visit, with the reference method given first. The reliability of the Choline-QFFQ was tested by comparing the results of three administrations of the questionnaire. The validity of the Choline-QFFQ was tested using a dietary reference method (three repeated 24-hour recalls). 24-hr dietary recall interviews were administered using the multiple-pass method [28] and pictures and portion size props provided by the DAEK (Steyn & Senekal 2004). Hand-written dietary records were reviewed by a U.S. registered dietician/research scientist (L.B.) who entered these data into the FoodFinder3**®** software program. The two interviewers and L.B. held regular Skype**®** meetings to discuss the interviews, questions that arose, and any outliers found.

Since FoodFinder3**®** does not provide values for choline content, a database was created to estimate the choline content of foods consumed by the women in the study. All foods reported by women in 24-hr dietary recall interviews and any additional foods included in the Choline-QFFQ (total = 382 foods) were matched to preparation-specific food items in the USDA Database for the Choline Content of Common Foods (Release 2 [36]). Choline content was calculated by linking the nutrient database number used in the USDA choline database with that used in the USDA National Nutrient Database for Standard Reference. For 16 reported foods that were not listed in the USDA choline database and consisted of multiple food items, recipes were constructed and used to calculate choline content based on the quantity and content of individual ingredients. An additional 21 foods not listed in the USDA choline database were matched with nutritionally comparable foods, chosen by M.S. and R.C. based on similar content, energy, and appearance. One food, Maltabella (reported in two 24-hr recall interviews), was not included, as no appropriate substitution could be found. Total choline content was calculated for each 24-hr recall interview and each Choline-QFFQ. Values from the three 24-hr recall interviews for a given woman were then averaged to calculate usual daily intake, which was used as the reference method value [32]. Inadequate dietary choline intake was defined as average daily intake below the Adequate Intake level (AI) per the Dietary Reference Intake [19].

### Statistical analyses

Statistical analyses were performed using SPSS v.23 software (IBM, Chicago, IL, USA). Reliability and validity analyses were conducted separately for drinkers and abstainers/light-drinkers. Reliability of the Choline-QFFQ was assessed by comparing the daily dietary choline intake values between visits 1 and 2; visits 2 and 3; and visits 1 and 3, adapting the validity analyses and interpretations suggested by Lombard et al. [37]. Validity was assessed by comparing daily choline intake from the reference method (average of the woman’s three 24-hr recall interviews) with daily dietary intake from the QFFQ at visit 1, the average of visits 1-2, and the average of visits 1-3; the latter two comparisons were conducted to evaluate whether validity is better with repeated administration of the QFFQ. Group-level agreement was examined using the Wilcoxon signed-rank test and by examining the mean difference between intake values from repeated QFFQ applications as a percentage of the recall value. To assess potential bias, Spearman correlation coefficients were calculated between the mean of the intake values for the repeated QFFQ applications and the mean difference of the two values (from Bland-Altman analyses). As suggested by Kowalkowska et al. [38], we also calculated the Bland Altman Index, the percentage of subjects with values outside of the limits of agreement (LOA; defined as mean ± 1.96 SD), with < 5% being acceptable. Individual agreement was examined by calculating the percentage of individuals placed in the same tertile by each measure and the percentage placed in opposite tertiles (i.e., lowest tertile for one and highest tertile for the other) and by calculating a weighted kappa statistic. A kappa statistic was also calculated to assess agreement on whether a woman reported intake below the choline AI. Spearman correlation was used to assess the strength of agreement between visits at the individual level. Intraclass correlations and within-subject coefficients of variation for [39] dietary nutrient intakes were calculated using the method developed by Hertzmark and Spiegelman (https://www.hsph.harvard.edu/donna-spiegelman/software/icc9/).

## Results

### Sample characteristics

The majority of the mothers (89.8%) were 20-40 years of age, and drinkers were 2 years older than abstainers/light-drinkers on average (Table 1). Three-fourths (76.1%) of the women had attended high school, but only 12.2% completed high school. Drinkers had attended school almost 1 year less than abstainers/light-drinkers. Average daily energy intake was similar between drinkers and abstainers/light-drinkers and was below the 2400 kcal/day recommended during pregnancy. As we have previously reported, the majority of the women received prenatal iron/folic acid supplementation and reported good adherence, taking the supplement on most days [23]. Almost three-quarters (72.5%) had full-term pregnancies. Almost all women (94.7%) completed at least two interviews, and 64.6% completed three interviews. Heavy drinkers and abstainers/light-drinkers did not differ in number of visits. As expected, alcohol use was heavy among drinkers, who averaged 9.4 standard drinks per occasion on 2.4 days per week around time of conception and 8.4 standard drinks on 1.3 days per week across pregnancy. All but 8 controls (9.6%) abstained from alcohol use: 4 reported drinking < 2 drinks per occasion (three of these reported 3 or fewer occasions, one 1-2 occasions per week); 2 drank < 3 drinks per occasion 1-2 times per month; and 2 reported a single binge episode that occured following recruitment into the study.

**Table 1:** Sample characteristics

	Heavy drinkers					Abstainers/light drinkers					*p* ^a^
	*N*	*M*	SD	*n*	%	*N*	*M*	SD	*n*	%	
Maternal age at conception (yr)	122	27.7	5.7			83	25.5	4.8			.004
Gravidity (no.)	122	2.9	1.7			83	2.5	1.3			.109
Marital status (no. married)	122			34	27.6	83			34	41.0	.046
Education (yr school completed)	122	9.3	1.7			83	10.0	1.6			.005
Average daily energy intake (kilocalories/day)	122	2247.9	810.1			83	2343.0	857.7			.421
Received prenatal iron/folic acid supplementation	119			104	87.4	80			69	86.3	.814
Takes supplements most days (supplemented only)				104	90.4				65	93.2	.367
Number of visits	122	2.6	0.7			83	2.5	0.7			.110
Weeks gestation											
Initiation of antenatal care	122	17.3	5.9			83	18.7	6.0			.111
Visit 1	122	22.9	5.8			83	25.5	5.0			.001
Visit 2	116	27.0	5.6			78	29.9	5.0			<.001
Visit 3	78	32.5	4.0			46	34.0	3.9			.050
Delivery	122	38.8	2.1			83	39.0	2.2			.626
Alcohol and drug use											
AA/day (oz)	122	0.9	1.2			83	0.0	0.0			<.001
AA/drinking day (oz)	122	4.2	2.4			83	0.2	0.5			<.001
Drinking days/wk (days)	122	1.3	1.1			83	0.0	0.1			<.001
No. reporting cigarette smoking	122			106	86.2	83			57	68.7	.002
Cigarettes/day (smokers only)		6.8	4.1				6.1	5.9			.435
No. reporting marijuana use	122			29	23.6	83			8	9.6	.011
Marijuana use (users only; days/mo)		9.7	9.4				4.0	4.7			.026
No. reporting methamphetamine use	122			12	9.8	83			15	18.1	.083
Methamphetamine use (users only; days/mo)		4.5	5.4				8.8	8.0			.119

AA = absolute alcohol; 1 oz AA ≈ 2 standard drinks

^a^From χ2 for categorical variables and *t*-tests for all continuous variables except for weight, BMI, triceps and biceps skinfolds, and MUAC, for which values from repeated measures regression models are presented; weight, BMI and MUAC models include control for weeks gestation at time of measurement

### Dietary choline intake

A large majority of women in both the heavy drinking and abstaining/light-drinking groups reported choline intake below the AI (Table 2). Both heavy drinkers and abstainers/light-drinkers in the cohort reported a lower choline intake at each consecutive visit on both the 24-hr recall interviews and the QFFQs, but this decrease over time was only statistically significant on the QFFQ. We have previously reported that average daily choline intake and the prevalence of inadequate intake were similar between heavy drinkers and abstainers/light-drinkers [23].

**Table 2:** Daily choline intake values by 24-hr recall interviews and semi-quantitative food frequency questionnaire (QFFQ)

	Intake below AI	Average daily intake (mg)	
	*n* (%)	Mean (SD)	Median (IQR)
Heavy drinkers			
24-hr recall^a^			
Visit 1 (*n* = 122)	95 (77.9)	338.6 (257.1)	275.4 (156.7 – 410.5)
Visit 2 (*n* = 116)	96 (82.8)	310.8 (221.0)	257.6 (176.1 – 359.4)
Visit 3 (*n* = 86)	73 (84.9)	279.8 (185.8)	229.1 (141.3 – 370.0)
Visits 1-2 (mean; *n* = 116)	94 (81.0)	327.5 (200.0)	274.7 (194.6 – 409.5)
Visits 1-3 (mean; *n* = 86)	73 (84.9)	304.6 (147.6)	273.9 (195.7 – 379.3)
QFFQ^b^			
Visit 1 (*n* = 121)	97 (80.2)	340.4 (205.1)	293.0 (208.2 – 436.0)
Visit 2 (*n* = 112)	100 (89.3)	286.0 (160.5)	267.9 (176.7 – 352.0)
Visit 3 (*n* = 78)	67 (85.9)	259.1 (160.5)	259.1 (175.9 – 337.4)
Visits 1-2 (mean; *n* = 112)	97 (86.6)	312.1 (152.1)	301.8 (203.3 – 386.9)
Visits 1-3 (mean; *n* = 78)	70 (89.7)	322.8 (180.5)	288.8 (186.3 – 410.5)
Abstainers/light drinkers			
24-hr recall^a^			
Visit 1 (*n* = 83)	70 (84.3)	336.8 (255.9)	279.7 (182.5 – 406.1)
Visit 2 (*n* = 78)	61 (78.2)	305.7 (179.7)	267.1 (151.8 – 416.7)
Visit 3 (*n* = 47)	39 (83.0)	285.9 (175.7)	232.2 (164.6 – 348.3)
Visits 1-2 (mean; *n* = 78)	66 (84.6)	318.1 (178.1)	282.6 (186.8 – 386.4)
Visits 1-3 (mean; *n* =47 )	39 (83.0)	303.1 (171.1)	247.6 (196.5 – 364.1)
QFFQ^b^			
Visit 1 (*n* = 83)	68 (81.9)	316.4 (215.1)	277.8 (166.5 – 378.5)
Visit 2 (*n* = 72)	62 (86.1)	275.3 (162.2)	279.0 (149.0 – 356.7)
Visit 3 (*n* = 42)	39 (92.9)	199.6 (113.5)	164.3 (125.1 – 211.9)
Visits 1-2 (mean; *n* = 72)	64 (77.1)	304.9 (178.0)	279.1 (175.7 – 402.9)
Visits 1-3 (mean; *n* = 42)	37 (88.1)	290.7 (169.6)	266.5 (161.6 – 371.2)

AI = adequate intake (450 mg choline/day)

^a^Paired t-test comparing values between visits: Drinkers: *p* = .191 for visits 1-2, .607 for visits 2-3, and .055 for visits 1-3; Abstainers/light-drinkers: *p* = .380 for visits 1-2, .702 for visits 2-3, and .318 for visits 1-3.

^b^Paired t-test comparing values between visits: Drinkers: *p* = .006 for visits 1-2, .921 for visits 2-3, and .019 for visits 1-3; Abstainers/light-drinkers: *p* = .224 for visits 1-2, .001 for visits 2-3, and .009 for visits 1-3.

### Reliability of the Choline-QFFQ

When examining the group-level reliability of the Choline-QFFQ among heavy drinkers between visits, results for percentage difference pointed to good agreement, while agreement reflected by the Wilcoxon signed rank test was poor (Table 3). In Bland-Altman analyses, 5% or fewer of values fell outside of the limit of agreement (LOA) when comparing visits 1 and 2 and visits 2 and 3, but just above the 5% cutoff when comparing visits 1 and 3. Bias was evident in the Bland Altman Spearman correlation analyses with a positive correlation between the difference in reported intake between the two visits and the mean choline intake value for the 2 visits. Results for individual-level tests showed that percentage classified in the same tertiles and opposite tertiles (agreement including chance) was good for all comparisons with the exception of visit 1 vs. visit 3, which was poor. Agreement excluding chance, as reflected by the weighted kappa statistic, and strength of agreement, reflected by the Spearman correlation coefficient, was acceptable-to-good for all comparisons. Results were almost identical among abstainers/light-drinkers for group- and individual-level tests. Values for the QFFQ within-subject coefficient of variation (.53) and the ratio of within- to between-subject variance (2.2) were similar to those for 24-hr recall interviews (.58 and 2.1, respectively).

**Table 3:** Reliability tests comparing dietary choline intake between quantitative food frequency questionnaire interviews

	Group-level				Individual-level					Summary	
	Agreement	Agreement	Agreement	Bias	Strength of association	Agreement including chance		Agreement excluding chance			
	Wilcoxon signed rank test *p*	Percentage difference	Bland-Altman LOA index	Bland-Altman Spearman correlation *p*	Spearman correlation^2^	Tertile classification		Kappa statistic		Group-level tests at “Good” or “Acceptable” (of 4 tests)	Individual-level tests at “Good” or “Acceptable” (of 5 tests)
						Same tertile	Opposite tertile	Weighted for tertiles	Above vs. below AI		
Heavy drinkers:											
Visit 1 vs. 2 (*n* = 112)	.001	M -0.4%	4.5%	< .001	.52	54.5%	9.8%	.44	< .001		
Interpretation^1^	Poor	Good	Good	Poor	Good	Good	Good	Acceptable	Poor	2	4
Visit 1 vs. 3 (*n* = 78)	.026	M -2.8%	7.7%	< .001	.43	47.4%	14.1%	.28	< .001		
Interpretation^1^	Poor	Good	Poor	Poor	Acceptable	Poor	Poor	Acceptable	Poor	1	2
Visit 2 vs. 3 (*n* = 78)	.606	M -18.4%	5.1%	< .001	.56	50.0%	9.0%	.41	< .001		
Interpretation^1^	Good	Acceptable	Good	Poor	Good	Good	Good	Acceptable	Poor	2	4
Abstainers/light-drinkers:											
Visit 1 vs. 2 (*n* = 72)	.522	M -7.1%	4.2%	.001	.42	56.9%	2.8%	.50	.001		
Interpretation^1^	Good	Good	Good	Poor	Acceptable	Good	Good	Acceptable	Poor	3	4
Visit 1 vs. 3 (*n* = 42)	< .001	M 9.1%	4.8%	< .001	.37	47.6%	2.4%	.49	< .001		
Interpretation^1^	Poor	Good	Good	Poor	Acceptable	Poor	Good	Acceptable	Poor	2	3
Visit 2 vs. 3 (*n* = 42)	.014	M 16.2%	2.4%	< .001	.50	50.0%	4.8%	.47	< .001		
Interpretation^1^	Poor	Acceptable	Good	Poor	Good	Good	Good	Acceptable	Poor	2	4

^1^Interpretation criteria [37]:

Wilcoxon signed rank test: Good: *p* > 0.05; Poor: *p* ≤ 0.05

Percentage difference: Good: 0.0 – < 11%; Acceptable: 11 - 20.0%; Poor: > 20%

Bland-Altman LOA index (% outside of limit of agreement): Good: ≤ 5%; Poor: > 5%

Bland-Altman Spearman correlation coefficient: Good: *p* > 0.05; Poor: *p* ≤ 0.05

Spearman correlation coefficient: Good: ≥ 0.50; Acceptable: 0.20 – 0.49; Poor < 0.20

Tertile classification (% in same tertile): Good: ≥ 50%; Poor: < 50%

Tertile classification (% in opposite tertile): Good: ≤ 10%, Poor: > 10%

Weighted Kappa statistic: Good: ≥ 0.60; Acceptable: 0.20 – 0.59; Poor: < 0.20

^2^*p* < .001 for all Spearman values

### Validity of the Choline-QFFQ

Among drinkers, agreement was good-to-acceptable on most group-level tests, with the exception of percentage difference between QFFQ values at visit 1 and the mean of the 24-hr recall interviews (reference method) and the LOA index when averaging QFFQ values from visits 1-2 (Table 4). Individual-level tests among drinkers were good for the Spearman correlation, acceptable for the weighted kappa test, and poor for both tertile classification methods (46.6% vs. ≥ 50% cutoff and 10.3% vs. ≤10% cutoff). Among abstainers/light-drinkers, all group-level tests were acceptable-to-good except for Bland-Altman Spearman correlation when using only visit 1 QFFQ values, with a weak, positive bias seen. At the individual-level, all tests were acceptable-to-good except for same-tertile classification when using the mean of visits 1-3, which was poor.

**Table 4:** Validation tests comparing dietary choline intake between the semiquantitative food frequency questionnaire and 24-hour recall interviews

	Group-level agreement				Individual-level agreement					Summary	
	Agreement	Agreement	Agreement	Bias	Strength of association	Agreement including chance		Agreement excluding chance			
	Wilcoxon signed rank test *p*	Percentage difference	Bland-Altman LOA index	Bland-Altman Spearman correlation *p*	Spearman correlation^2^	Tertile classification		Kappa statistic		Group-level tests at “Good” or “Acceptable” (of 4 tests)	Individual-level tests at “Good” or “Acceptable” (of 5 tests)
						Same tertile	Opposite tertile	Weighted for tertiles	Above vs. below AI		
Heavy drinkers:											
Visit 1 (*n* = 121)	.258	M 30.8%	5.0%	.11	.39	48.8%	14.9%	.26	.21	3	3
Interpretation^1^	Good	Poor	Good	Good	Acceptable	Poor	Poor	Acceptable	Acceptable		
Visits 1-2 (mean) (*n* = 116)	.976	M 16.6%	8.0%	.552	.39	46.6%	10.3%	.31	.20	3	3
Interpretation^1^	Good	Acceptable	Poor	Good	Acceptable	Poor	Poor	Acceptable	Acceptable		
Visits 1-3 (mean) (*n* = 78)	1.00	M -9.5%	5.1%	.148	.43	47.4%	7.7%	.40	.18	4	2
Interpretation^1^	Good	Good	Good	Good	Acceptable	Poor	Poor	Acceptable	Poor		
Abstainers/light-drinkers:											
Visit 1 (*n* = 83)	.440	M 7.2%	4.8%	.04 (.23)	.51	50.6%	9.9%	.39	.35	4	5
Interpretation^1^	Good	Good	Good	Poor	Good	Good	Good	Acceptable	Good		
Visits 1-2 (mean) (*n* = 68)	.366	M 0.0%	4.2%	.269	.52	50.0%	8.8%	.42	.35	4	5
Interpretation^1^	Good	Good	Good	Good	Good	Good	Good	Acceptable	Acceptable		
Visits 1-3 (mean) (*n* = 42)	1.00	M 5.2%	2.4%	.171	.56	45.0%	10.0%	.33	.63	4	4
Interpretation^1^	Good	Good	Good	Good	Good	Poor	Good	Acceptable	Good		

^1^Interpretation criteria [37]:

Wilcoxon signed rank test: Good: *p* > 0.05; Poor: *p* ≤ 0.05

Percentage difference: Good: 0.0 – < 11%; Acceptable: 11 - 20.0%; Poor: > 20%

Bland-Altman LOA index (% outside of limit of agreement): Good: ≤ 5%; Poor: > 5%

Bland-Altman Spearman correlation coefficient: Good: *p* > 0.05; Poor: *p* ≤ 0.05

Spearman correlation coefficient: Good: ≥ 0.50; Acceptable: 0.20 – 0.49; Poor < 0.20

Tertile classification (% in same tertile): Good: ≥ 50%; Poor: < 50%

Tertile classification (% in opposite tertile): Good: ≤ 10%, Poor: > 10%

Weighted Kappa statistic: Good: ≥ 0.60; Acceptable: 0.20 – 0.59; Poor: < 0.20

^2^*p* < .001 for all Spearman values

## Discussion

In this prospective longitudinal cohort of heavy drinking pregnant women and abstainers/light-drinkers, we developed and demonstrated reliability and validity of a QFFQ that assesses average daily choline intake and requires minimal time (~10-15 minutes) and resources (food identification cards and portion estimation props) to administer. To our knowledge, this is the first Choline-QFFQ to be developed and validated for use with pregnant women and the first to be used in the Cape Coloured community in South Africa. While this choline quantitative FFQ presented is specific to the Cape Coloured population, the development and validation steps we employed may be used to develop population-specific choline quantitative screening FFQs in other populations. Furthermore, the Choline-QFFQ performed well in our study population, suggesting that a Choline-QFFQ (and the development and validations steps we employed) can be used even in populations in which education is poor and/or socioeconomic status low. Despite extensive prevention efforts and guidelines (ACOG, 2011) and 4 decades of scientific research demonstrating teratogenic effects of prenatal alcohol exposure, women worldwide continue to drink during pregnancy. FASD comprise the most common preventable cause of neurodevelopmental disabilities, with prevalence estimates of 1.1-5.0% in the US and Western Europe [40, 41] and 13.6-20.9% in South Africa [9]. Evidence from both animal [10, 11, 42, 43] and human [14, 15] studies has demonstrated that high-dose choline supplementation can mitigate many of the teratogenic effects of alcohol. It is thus of concern that over 80% of the both heavy drinkers and abstainers/light-drinkers in this study reported choline intake below the AI in all assessments, as has been reported in other populations such as the U.S. [25], Canada [26], and New Zealand [27]. Given our reliability and validity findings, the QFFQ developed in this study may be used to screen alcohol-using pregnant mothers in Cape Town to identify women who may benefit from choline interventions, whether they be dietary counseling or choline supplementation programs. Given our finding that abstaining/light-drinking women in this cohort had similarly high rates of dietary choline inadequacy, this QFFQ may also be used to identify non-drinking pregnant women at risk for choline deficiency, which poses its own potential harms to the mother and developing fetus, including neural tube and craniofacial defects, as well as possible effects on neurocognitive development [18, 24, 44, 45].

The steps of development and validation we employed can be used to develop population-specific choline quantitative screening FFQs in other populations. Development and validation of a nutrient-specific screening QFFQ involved several steps, including 1) development of the local food list; 2) determining the most appropriate recall period, frequency options and portion size estimation method(s), based on the aim of the dietary intake assessment, the target population, and time available for the administration; 3) identifying an appropriate reference method; and 4) identification of interpretable statistical tests for examining the reliability and validity of the newly developed QFFQ. The food list for our Choline-QFFQ was developed based on the integration of the best known dietary sources of choline and knowledge of commonly consumed foods in the Cape Coloured community in Cape Town by a panel of nutrition researchers with expertise on the dietary patterns of the target population. The recall period was set at the previous week based on recommendations by Willett [28]. Portion size estimation was based on portions used in the FoodFinder program, which was developed for use in this population, and modified based on pilot interviews with women from this community. Administration of the Choline-QFFQ using the picture sort method proved to be simple and quick (~10-15 minutes), which is ideal for implementation as a screening procedure in a community healthcare setting. The dietary reference method used was three 24-hour recall interviews, the method most strongly recommended for validation of food frequency questionnaires [32], especially in communities where literacy and education issues may preclude the use of food diaries, such as this socioeconomically disadvantaged community in Cape Town.

We used the statistical tests and interpretations recommended by Lombard et al. [37] to assess different features of reliability and validity. Lombard recommends three group-level comparison tests (comparison of means using the Wilcoxon signed rank test, percentage difference, and Bland Altman Spearman test for bias) and four individual-level tests (Spearman correlation coefficient, same- and opposite-tertile classification, and the weighted kappa statistic for tertile classification). We added a fourth group-level test, the Bland Altman limit of agreement index (as recommended by Kowalkowska et al. [38]), and a fifth individual-level test, a kappa statistic examining the classification agreement for whether a woman reported intake below the choline AI, to provide a measure of clinical relevance. Group level tests are most relevant for research studies, where errors for a single woman are less important as long as group-level associations are acceptable-to-good, and individual-level tests are most relevant for clinical practice, in which a woman’s reported values will guide targeted recommendations and/or interventions. The majority of the five individual level statistical tests indicated acceptable to good reliability of the QFFQ for all comparisons conducted for both heavy drinkers and abstainers/light-drinkers, except for comparisons of visits 1 and 3 among drinkers, suggesting that reliability may decline over time. At the group level, reliability was good-to-acceptable for 2 of 4 tests, namely percentage difference in reported values between visits (≤ 20% = acceptable) and the Bland Altman limit of agreement index, a measure of how many outliers are present. Reliability was poor for the Wilcoxon signed rank test, a measure of how similarly ranked women are between visits, and the Bland Altman test for bias, which demonstrated a greater difference between reported intake between visits if the reported intake was higher. When examining the validity of the Choline-QFFQ compared with the mean of three 24-hour recalls, all group-level tests were acceptable-to-good, as were all individual level test results, except for classification into tertiles for the alcohol users for all comparisons conducted. Of note, the weighted kappa statistic for tertile classification was acceptable across almost all comparisons. The weighted kappa assesses agreement excluding chance and thus may provide a more accurate measure of agreement than the raw tertile agreement % measures.

Reliability and validity are measured separately, but results of the tests should be interpreted in light of each other. Although the QFFQ demonstrated both reliability and validity, tests of validity were more consistently acceptable-to-good than tests of reliability, particularly at the group level, as reflected in the Wilcoxon and Bland Altman bias tests, and the individual-level, when examining agreement as to whether a woman had inadequate intake. The relatively poorer reliability seen in these tests is likely due to the fact that women reported lower choline intake at each visit. This trend may reflect interview fatigue, as has been reported in several other studies [46]; after familiarization with the procedure, women may have chosen fewer cards from the QFFQ card sorting as a result of fatigue and/or a conscious attempt to shorten the interview. Indeed, reliability was better from visits 1-2 than 2-3, but this difference may also reflect the longer time period between interviews (8 weeks for visits 2-3 vs. 4 weeks for visits 1-2). An actual reduction in food and thus choline intake towards the end of the pregnancy must, however, also be considered. The mean energy intake per day decreased somewhat from each visit to the next (2294 kcal/day at visit 1, 2280 kcal/day at visit 2, 2194 kcal/day visit 3). As repeated administration of the QFFQ did not significantly improve validity the Choline-QFFQ screener could be used as a single-visit screener.

This study had limitations common to other longitudinal studies of nutrition. 24-hr dietary recall interviews can yield inaccurate estimates of usual intake due to recall errors. However, we have previously reported that intraclass correlations and within-subject coefficients of variation for dietary nutrient intakes in this cohort, including choline, were similar to those of NHANES and other peer-reviewed epidemiologic studies in the U.S. [23], indicating that random error in this study did not exceed levels generally accepted in the nutritional epidemiology community. Furthermore, we found that energy intake predicted gestational weight gain, further supporting the validity of the 24-hr recall data. Where possible, the method of triads, in which two dietary assessment methods and a biochemical marker of nutritional status for a given nutrient are compared, is recommended for validation of FFQs [46]. We did not employ this method because free choline and other choline metabolites are tightly regulated and relatively unresponsive to dietary changes, as homeostatic mechanisms, such as estrogen-induced endogenous choline production by the PEMT enzyme during pregnancy act to keep plasma choline values in the normal range; lack of correlation between diet and biochemical values is thus common, particularly in cohorts without high rates of severe choline deficiency [47, 48]. Differences between true and estimated levels of maternal alcohol consumption are likely small, given the validity of the interviewing techniques demonstrated in this community in relation to meconium levels of fatty acid ethyl ester metabolites of alcohol [49], infant and child behavior [7, 35, 50], somatic growth [51], and brain structure [52–54] and function [55].

## Conclusions

To our knowledge, this is the first quantitative choline food frequency screening questionnaire to be developed and validated for use in both heavy and non-drinking pregnant women and the first to be used in the Cape Coloured community in South Africa. Given the high prevalence of inadequate choline intake in this and other communities worldwide and the growing evidence that maternal choline supplementation may benefit infant neurodevelopment and can mitigate adverse effects of prenatal alcohol exposure, this tool may be useful for both research and for future clinical outreach programs aimed at identifying pregnant women at risk for choline deficiency. The Choline-QFFQ development and validation methodology we employed can be used in other communities as well, which may be of significant public health utility given the widespread public health burdens of dietary choline inadequacy and maternal alcohol consumption during pregnancy. Our findings demonstrating validity of the QFFQ for use in this highly disadvantaged, poorly educated population supports its potential utility for use in a broad range of social contexts.

## Abbreviations

AA: Absolute alcohol
FAS: Fetal alcohol syndrome
FASD: Fetal alcohol spectrum disorders
QFFQ: Quantitative food frequency questionnaire
